# Prevalence of Evidence-Based School Meal Practices and Associations with Reported Food Waste across a National Sample of U.S. Elementary Schools

**DOI:** 10.3390/ijerph18168558

**Published:** 2021-08-13

**Authors:** Hannah G. Calvert, Punam Ohri-Vachaspati, Michaela McQuilkin, Peter Boedeker, Lindsey Turner

**Affiliations:** 1 Center for School and Community Partnerships, College of Education, Boise State University, 1910 University Drive, Boise, ID 83725, USA; hannahcalvert898@boisestate.edu (H.G.C.); michaelamcquilkin@boisestate.edu (M.M.); 2 College of Health Solutions, Arizona State University, Phoenix, AZ 85004, USA; Punam.Ohri-Vachaspati@asu.edu; 3 Department of Curriculum, Instruction and Foundational Studies, College of Education, Boise State University, 1910 University Drive, Boise, ID 83725, USA; peterboedeker@boisestate.edu

**Keywords:** breakfast, lunch, recess, meal duration, promotional strategies, nutrition policy

## Abstract

Providing meals at school is an important part of the hunger safety net for children in the United States and worldwide; however, many children do not receive school meals even when they qualify for federally-subsidized free or reduced-priced meals. This study investigates the prevalence of several evidence-based practices that have previously been shown to increase the reach and impact of school meals. A survey was sent to a national sample of US elementary schools, with items examining practices regarding school breakfast, school lunch, recess, the promotion of meals, nutrition standards, and food waste, during the 2019–20 school year. Almost all schools that offered lunch also offered breakfast. More than 50% used a breakfast service strategy other than cafeteria service, such as grab-and-go breakfast meals. Providing at least 30 min for lunch periods and providing recess before lunch were reported by less than half of schools. About 50% of schools reported using only one or fewer meal promotional strategies (such as taste tests) throughout the school year. Use of more promotional strategies was associated with less reported food waste in a multivariable regression model accounting for school demographic characteristics. Findings show that some evidence-based practices for school meals are being implemented, but many recommendations are not being widely adopted.

## 1. Introduction

The National School Lunch Program (NSLP) and the School Breakfast Program (SBP) are the two largest school nutrition programs administered by the United States Department of Agriculture (USDA). They are both a crucial part of the food security safety net for children and adolescents in the United States [1], with roughly 30 million students at public schools across the United States (US) receiving meals through the NSLP each day [2], and about 22 million through the SBP. Having access to nutritious meals at school has several benefits for students, including improved behavior, academics, and overall nutrient intake [3,4,5,6]. These benefits come at low or no cost to many families, as more than 70% of students who participate in the NSLP receive meals for free or at a reduced price based on household income [2]. Food insecurity rates among children in the US were 6.5% in 2019 [7] and were estimated to have more than doubled in 2020 during the COVID-19 pandemic [8,9,10], showing that there is still a need to increase the reach of assistance programs.

### 1.1. Evidence-Based School Meal Practices and Their Prevalence in US Schools

Over the last decade, schools across the US have implemented several evidence-based practices intended to improve meal participation and consumption among students. These practices can increase program reach by reducing financial and logistical barriers to accessing meals as well as increasing student and family interest in school meals [11]. The SBP, in particular, has seen a large increase in student participation from innovations, such as offering flexible options for students to eat breakfast at school, such as grab-and-go breakfasts, or serving breakfast in the classroom [12,13,14]. These strategies lessen logistical and timing barriers related to students not arriving at school early enough to eat in the cafeteria before classes begin [15]. Nationally-representative data on US school districts from the School Health Policies and Practices Study in 2016 indicated that most districts did not have a policy about using alternative breakfast service strategies [16]. The 2014–15 USDA School Nutrition and Meal Cost Study showed that traditional breakfast delivery was still the predominant service model [17]. For the NSLP, practices such as offering recess before lunch and having a minimum of 20 min of seated time for all students to eat their meal increase meal consumption [18,19,20]. However, policies for adequate seated time at lunch and recess before lunch are estimated to be in place at 40% of schools and 8% of schools, respectively [16], with most schools offering 30 min in total for lunch, and recess after lunch, based on recent national US data [17].

Schools also use a variety of promotional strategies to increase interest in school meals. The USDA cites involvement of administrators, teachers, and parents in promoting school meals (such as through parent meetings and school newsletters) as important strategies for increasing the reach of school meal programs [11]. Reviews of fruit and vegetable promotion interventions for children and adolescents conclude that hands-on strategies which encourage active participation in meal preparation, and involvement of parents and school staff, are effective for increasing students’ fruit and vegetable consumption at school meals [21,22]. These strategies may include taste tests [23,24], or having a professional chef train food service staff in preparing healthier meals [25]. Despite the benefits of these approaches, the reported prevalence of such strategies was relatively low in schools throughout the US, as of school year 2014–15 [26,27].

### 1.2. Evidence-Based Meal Practices and Associations with Food Waste

Best practices in NSLP and SBP implementation and meal delivery not only affect reach of these programs in terms of how many students participate, and effectiveness in terms of improving diet and reducing food insecurity, but they also have implications for food waste, which is an important economic and environmental concern faced by school food programs [28]. Studies using weighing and digital photography methods of measuring plate waste show that, for school lunches served, roughly one third to one half of vegetables and fresh fruit are thrown away [28,29]. However, food waste can be decreased by longer lunch periods that allow students more time to consume meals; less food waste occurs with 30 min versus 20 min lunch periods [30]. Furthermore, longer lunch periods support a higher intake of important nutrients [19,30,31,32], and reduce students’ hunger and increase social-emotional well-being [33]. Offering midday recess prior to the lunch period is also beneficial; one study found that having recess directly before lunch decreased overall waste by 10% [34], while others have shown benefits for milk consumption [35,36], and fruit [18,20] and vegetable [20] consumption. Several studies have explored how interventions can reduce food waste [25,37], but none have quantified how schools’ use of multiple promotional strategies may affect food waste.

### 1.3. Regulatory Rollbacks and Evidence-Based Meals Practices

Optimizing the nutritional content of school meals based on scientific evidence has always been a high priority for the USDA. As directed by the Healthy, Hunger-Free Kids Act of 2010, the USDA updated school meal patterns and nutrition standards [38], increasing the amount and variety of fruits and vegetables, requiring that at least half of all grains served be wholegrain-rich (increasing to 100% by 2014), and limiting added sugars, sodium, and saturated fat. Meal quality improved [39], as did diet and weight outcomes of participating students [40,41] after the implementation of these standards. Despite concerns about healthier options being less enticing to students, food waste [42,43,44] remained stable after healthier meal standards were phased in during 2012–2013, and there was no evidence of substantial decreases in the number of students taking school meals [45,46]. In 2018, however, nutrition standards were rolled back, reducing the amount of whole grains required to be served back to 50%, delaying reduced sodium standards, and allowing 1%-fat, flavored milk. Subsequently, in 2020 a federal court ruling struck down the flexibilities. Information about school meal practices during this time is sparse, with little known about whether schools utilized rollbacks during the short window in which they were available.

### 1.4. Study Purpose

Periodic surveillance of the prevalence of evidence-based school meal practices is important for evaluating change and for targeting technical assistance and resources. There have not been nationally-representative US data gathered about school meal practices since the School Health Policies and Practices Study in 2014 and the School Nutrition and Meal Cost Study in 2014–15, and studies examining the relationship between such practices and food waste are limited. The current research explores the prevalence of school meal practices. Using cross-sectional data from a nationally-representative survey of elementary schools in the 2019–20 school year, the aims were: (1) to examine the prevalence of various evidence-based practices related to school meals; and (2) to examine associations between school lunch practices and reported estimates of food waste in school lunches.

## 2. Materials and Methods

Data were obtained via survey of a national sample of elementary schools during the 2019–2020 school year. Survey sampling methodologists at the Institute for Social Research at the University of Michigan originally developed the sample frame and analytic weights for a study conducted by the senior author (L.T.) in school year 2013–14, which also examined school nutrition and wellness practices. The sample was selected through stratified simple random sampling, designed to be nationally representative of US public elementary schools (i.e., schools with a grade 3 class). Due to differing grade enrollments for schools across the country (i.e., kindergarten to grade 4; grades 1–5, etc.), those serving at least one grade 3 class (students typically 9 years of age) were defined as elementary schools for this study. Two stages were used for selection: first, a nationally representative sample of districts was developed based on state location, urbanicity, and number of students; second, schools were selected within each district group, with the measure of size for selection of schools based on the number of grade 3 students. Additional details are available elsewhere [47].

### 2.1. Data Collection

On 26 August 2019, the research team mailed surveys and invitation letters to the principals of the sample of 1,010 eligible schools. The invitation letter to principals detailed the background and purpose of the survey, and suggested delegation of survey completion to another staff member at the school knowledgeable about wellness practices, if needed. Respondents were given the option of completing the mail-back survey with the provided return postage, or using a Qualtrics link to complete it online. The research team gave several prompts to schools to complete the survey, including emails, phone calls, and a second mailed survey. The survey closed on 28 February 2020 with a 55.3% response rate and 559 respondents (377 completing online and 182 responding via mail). Surveys were filled out by the principal at 274 schools and by other school personnel in the remaining 285 schools. The roles of these respondents included teachers (n = 107), office/business managers (n = 70), nurses (n = 39), cafeteria staff (n = 5), other roles (e.g., counselors, administrative assistants; n = 31), or responses were missing (n = 33). Respondents were given the option of receiving a $50 online gift card as compensation for their time filling out the survey. This study was approved by the Institutional Review Board at Boise State University (protocol no. 101-SB19-151).

### 2.2. Measures

The full survey included 66 items about school policies and practices related to physical education, physical activity, nutrition and school meals, school fundraisers, drinking water, and wellness teams. The current analysis describes results from 11 items regarding school meals. Four of these items were used in a previous long-running school surveillance study conducted by two of the current authors (P.O.-V. and L.T.) [47,48], including items assessing schools’ participation in the SBP and NSLP, as well as duration of lunch, and timing of recess. Additional items were developed by the current authors to examine the prevalence of additional practices relevant to school nutrition, either based on the team’s prior research examining these topics, other research and policies assessing these practices, and our development of new items to examine time-sensitive issues such as national policy changes. While some of the items have previously been used, the newly-developed items for this study were not pilot-tested; for readers to assess face validity, the verbatim wording of each item is provided hereafter.

The first set of school meal items pertained to the School Breakfast Program, including, “Does your school participate in the USDA reimbursable School Breakfast Program?” with a yes/no response option and, “If yes, which of the following strategies are used to serve breakfast?” with check-all-that-apply options of: (a) traditional breakfast service in the cafeteria before class; (b) breakfast in the classroom; (c) second-chance breakfast; and (d) grab-and-go breakfast. The remaining items asked about school lunch, starting with, “Does your school participate in the USDA reimbursable National School Lunch Program?” (yes/no). While other items did not specify particular grade levels, the two items about lunch duration and timing were framed specific to third grade students, due to common practices at schools where schedules differ by grade. The item about lunch duration asked: “How long does each student have to eat lunch, not including recess? If lunch is combined with recess, please estimate how many minutes are generally set aside for lunch, for third grade students.” Responses were open-ended. During data cleaning, three groups were developed, based on recommended practices for meal duration: 20 or fewer minutes (coded = 1), 21 to 29 min (coded = 2), or 30 or more min (coded = 3). The item about lunch duration asked: “Please indicate the timing of lunch in relation to mid-day recess, for third grade students,” with four categorical response options: third grade students have lunch and then go directly out for recess (coded = 1), third grade students have recess and then come in for lunch (coded = 2), third grade students do not have recess directly prior to or after lunch (coded = 3), or varies by class (coded = 4). 

Items regarding food waste were developed by the research team, specifically for this study. Two items inquired about lunch waste. The first was worded as: “Some schools find that students throw away a lot of the lunches that are served in the cafeteria, whereas others do not. To what extent does “food waste” occur for the lunches served at this school?” with response options of very much (coded = 1), somewhat (coded = 2), a little (coded = 3) or not much (coded = 4). A subsequent item asked, “Considering the school lunch, (not lunches brought from home), how much of each of the following items would you estimate students typically eat, as compared to what is thrown away?” Meal components assessed were: (1) entrée/main course; (2) fruit; (3) vegetable; and (4) milk. Response options for each component were: all (coded = 1), most (coded = 2), some (coded = 3), a little (coded = 4), or none (coded = 5).

In addition, two items were developed by the current authors for an exploratory assessment of schools’ use of the regulatory rollbacks for school meals issued in 2018, worded as: “In 2018, schools were allowed to use new ‘relaxed’ nutrition standards to change the types of foods and beverages offered in school lunches. To your knowledge, has your school district used this flexibility to change anything about school meals since last year?” with response options of yes (coded = 1), no (coded = 2), or don’t know (coded = 3). The second item asked “Compared to this time last year, do lunches at this school offer less, the same, or more of the following?” with rows for responses about: (1) amount of fruits and vegetables; (2) variety of fruits and vegetables; (3) whole grain options; (4) low fat dairy products; and (5) variety of entrée options. Response options were: less or fewer (coded = 1), same (coded = 2), or more (coded = 3). 

The last item asked about school meal promotion, with the stem of “Has your school used any of the following strategies to promote healthier lunches during the past year?” followed by a list of specific practices that had previously been examined by the current researchers, using data from other national surveillance studies [26,27]. The strategies were: (1) student taste tests; (2) student advisory groups; (3) cooking club demonstrations/classes; (4) social media (Facebook, Twitter, etc.); (5) engagement with parent groups; (6) and newsletters. For each strategy, response options were never (coded = 0), once or twice (coded = 1), or three or more times (coded = 2)

### 2.3. Demographic Data

School demographic information was obtained using the publicly-available Common Core of Data from the National Center for Education Statistics (NCES) [48]. US census region was classified for each school (Northeast, Midwest, South, and West). Locale classifications included urban, suburban, town, or rural, based on the NCES urban-centric locale codes [48]. School size, racial/ethnic composition, and socioeconomic composition were used as covariates in statistical models, and were coded in categories similar to the authors’ prior work [49]. Total student enrollment was used to reflect school size, which was coded in three groups (≤450, 451 to 621, and >621). The racial/ethnic composition of each school’s student body was coded into one of four exhaustive and exclusive categories: predominantly (≥66%) White non-Latino, majority (≥50%) Black non-Latino, majority (≥50%) Latino, and other (diverse, or majority Asian or Native American). School-level socioeconomic status (SES) was proxied by the percentage of students eligible for free/reduced-priced lunch (FRPL), coded as higher-SES (≤33% eligible), moderate-SES (>33% to ≤66% eligible), and lower-SES (>66% eligible). 

### 2.4. Data Analysis

Paper surveys were double-entered into Qualtrics, downloaded, and compared for data entry errors. Once paper survey responses had been confirmed, they were merged with online survey responses, and demographic data were merged. Of the 559 schools, 17 were dropped from analysis due to not participating in the NSLP. Among the included 542 schools, 29 were missing data for FRPL. In these cases, the last known value (i.e., FRPL percentage from the previous school year) was used to fill in missing data. There were no missing values for other demographic variables.

#### 2.4.1. Weighting

Sampling weights were included with the original sampling frame, developed to allow for national inferences. These weights accounted for the probability of selection and school size (number of grade 3 students); more details are available elsewhere [47]. Post-response calibrations were made to the weights, to account for propensity to respond in 2019–20, using school characteristics associated with response status (yes/no).

#### 2.4.2. Statistical Analysis

Demographic characteristics of schools and survey item responses were tabulated with frequencies and weighted percentages. Survey responses had small amounts of missingness (ranging from 3.1% to 8.1% of cases missing per item). The svyset and svy commands were used in the STATA software package to account for the survey design, including clustering of schools within district, stratification, and weighting. To examine the prevalence of meal practices in 2019–2020, weighted prevalence values with 95% confidence intervals were computed.

Linear regressions estimated the associations between school meal practices and lunch waste. For parsimony, composite variables were created where possible, with assessment of inter-item reliability using Cronbach’s alpha. School lunch waste variables generated an α = 0.665 between the four items (entrée, milk, fruit, and vegetable waste), which improved to 0.729 with the removal of the vegetable item. A composite was created by averaging the three items, while vegetable waste was considered a separate outcome. Linear regression models included all covariates (FRPL, race/ethnicity, school size, region, and locale), with separate models for the outcomes of overall food waste, a composite waste variable, and vegetable waste. Analyses were completed using STATA SE version 16.1 (StataCorp LP, College Station, TX, USA).

## 3. Results

### 3.1. Descriptive Statistics and Prevalence Estimates for School Meal Practices

Demographic characteristics for all schools in the sample are provided in Table 1.

Descriptive statistics for survey items pertaining to school meal practices in 2019–20 are presented in Table 2. For questions about school breakfast service practices, the sample was restricted to those that responded “yes” to indicate that the school participates in the SBP (n = 476, 87.8% of the full sample). The alternative breakfast service style most commonly offered in combination with traditional/in cafeteria breakfast was grab-and-go breakfast (74 schools, 15.5%), followed by breakfast in the classroom (49 schools, 10.3%), then second-chance breakfast (47 schools, 9.9%). The most commonly offered stand-alone strategy (when no in-cafeteria breakfast was served) was breakfast in the classroom (89 schools, 18.7%), followed by grab-and-go breakfast (42 schools, 8.8%), then second-chance breakfast (3 schools, 0.6%). 

The percentage of schools that have utilized flexibilities for nutrition standards in meals, and which practices were changed from the prior year, are shown in Table 3. Only 21% of respondents reported using flexibilities, but nearly half (49.5%) did not know. 

Table 3 shows the frequencies of school meal promotional strategies. The number of strategies used at least once or more times by each school was calculated, showing that the majority of schools used few, if any, strategies: 161 schools (33.3%) reported using no promotional strategies, 89 (19.4%) used one strategy, 90 (17.6%) used two strategies, 67 (13.4%) used three strategies, 49 (9.1%) used four strategies, 23 (4.1%) used five strategies, 15 (3.2%) used six strategies, and 48 did not respond.

For the single item asking respondents to indicate “To what extent does food waste occur for the lunches sold at this school?”, 14.7% (n = 78) indicated “very much,” 56.4% (n = 295) indicated somewhat, 21.9% (n = 114) indicated “a little,” and 6.9% (n = 35) indicated “none” (responses not shown in tables). For the subsequent item asking how much of each specific lunch components student eat, responses are detailed in Table 4.

### 3.2. School Meal Environment Characteristics Predicting Food Waste

Results of three multivariable linear regression models examining associations between respondent-reported food waste and school meal practices are shown in Table 5. For this model, total number of promotional strategies (used at least once per year) at each school was included as a predictor variable. In addition, lunch timing was a predictor (comparing recess-after-lunch with other scheduling), as was lunch duration (comparing schools with at least 30 min of lunch versus shorter lunch periods). All demographic covariates were included as controls. In the first model, the use of more promotional strategies was associated with less overall perceived food waste. In the second model, the use of more promotional strategies was associated with less waste, reflected by the school meal service waste composite variable. Additionally, having recess neither directly before or after lunch was associated with less waste, and schools located in towns reported less waste relative to schools in urban areas. In the third model, variables significantly associated with more vegetable waste included being a school with a majority Latino student population as compared to schools with a majority White student population, and being in suburban or rural areas compared to urban areas.

## 4. Discussion

This study assessed public US elementary schools’ reported use of several evidence-based nutrition practices such as flexible breakfast service strategies, providing adequate seated time for students to eat lunch, and offering recess before lunch—strategies that have been recommended for increasing the reach and effectiveness of school meal programs, and decreasing food waste in those programs [50]. We also examined the relationship between school meal practices and reported food waste in school lunches.

Our study found that among schools that participated in the NSLP, about 92% also participated in the SBP. This is similar to what was observed in the School Health Policies and Practices district-level data from 2016 [16]. In the current sample, while about 44% of schools reported serving breakfast using only traditional/in-cafeteria breakfast, half reported using other strategies for breakfast service. Grab-and-go breakfast was used most commonly—alongside traditional in-cafeteria breakfast—and breakfast in the classroom was used most frequently in schools that did not serve in-cafeteria breakfast. Compared to data from the US School Nutrition and Meal Cost Study in 2014–15, the use of several of these strategies seems to have increased, including breakfast in the classroom (27% in 2014–15 vs. 30% in our study), and grab-and-go breakfast (7% in 2014–15 vs. 23% in our study). The expansion of reported use of these breakfast service strategies is encouraging, as these more flexible methods have been shown to increase the reach of the SBP [12,13,14], allowing more students to receive meals who otherwise may receive little to nothing nutritious to eat at the start of the day.

For duration of lunch, 61% of respondents reported that their school’s third grade lunch period lasted less than 30 min. The US Centers for Disease Control and Prevention (CDC) recommends that students have 30 min scheduled for lunch, to provide at least 20 min of seated time for socializing and eating after students purchasing lunch get their meals [50]. Our data indicate that many schools are falling short of meeting the 30-min lunch time standard, and nearly 40% of schools are offering 20 min or fewer for total lunch time (not just seated time to eat). This is suboptimal, as research has shown that, compared to having 25 min for lunch, students consume significantly less of their entrée, milk, and vegetable selections when they have fewer than 20 min to eat [19]. Data from the CDC’s School Health Policies and Practices Study indicated that in 2016, only 40% of school districts recommended or required a minimum amount of time students are given to eat lunch after receiving their meal, and among those schools, about two-thirds of them set the minimum time at 20 min or more [16]. Schools in states with laws regarding lunch duration are more likely to provide students with a 30-min seated period for lunch [27], so having a policy regarding seated time is a potentially important step in the right direction.

Recess timing in relation to the lunch period has also been a focus of study, particularly as it relates to student eating patterns. Organizations in the US including the CDC and USDA recommend that schools schedule recess before lunch [50,51,52]; however, our data indicate that approximately one in four schools implement this practice consistently for their third grade classes. A 2017 survey of elementary school principals in the US state of Indiana found similar rates of offering recess before lunch, showing that it was implemented in 30.7% of schools [53]. District-level data from the nationally-representative School Health Policies and Practices Study in 2016 showed that about 30% of elementary school districts had requirements or recommendations related to offering recess before lunch, which also aligns with our findings that less than 30% implement this practice. Several studies have found that providing recess before lunch for elementary school students has benefits, including improved fruit and vegetable consumption [18,20,54], improved milk and overall energy consumption [35,36], reduced food waste, and better student behavior at lunch time [53,55]. While consistent effects are not seen across these studies for consumption of the various food groups, they generally favor associations between improved nutrient intake when recess occurs before lunch. Scheduling recess before lunch can be a challenge for school administrators, who navigate the complexities of scheduling several lunch periods while managing recess supervision and transition logistics [53,56]; however, the scheduling of recess before lunch is a no-cost strategy with some evidence for increasing healthy dietary consumption among children, and reducing food waste. Further, consuming more at lunch could lead to decreased afternoon hunger, improving student focus and academic performance for the rest of the day [57,58,59]. 

Current data show that school meal promotion strategies continue to be underutilized by elementary schools. In this study, a little over half of schools used none or only one promotional strategy throughout the school year. The most commonly used strategy was the school newsletter, followed by engagement with parent groups, and student taste tests. However, schools seldom reported using any strategy three or more times during the school year. Results from the USDA’s School Nutrition and Meal Cost Study in 2014–15 showed that about 64% of school food service staff reported using taste tests as a promotional strategy at their school, and 37% reported engaging with parent teacher associations or parent groups to discuss school meals [17]. Our data showed lower rates of both of these practices among schools in 2019–20, with 25% of schools reporting using taste tests at least one to two times per year, and 27% of schools reporting engaging with parent groups. Student taste tests have shown promise in increasing children’s intake of fruits, vegetables, and novel foods [23,24,60]. We found more frequent use of promotional strategies targeting parents, rather than those that directly communicate with students. Interventions can be effective in improving parent perceptions of school meals [61,62], but appropriate messaging and engagement, targeted toward the specific parent population and their values, must be considered. Food service staff report time and money as significant barriers to implementation of meal promotion strategies [60], and administrators also must see the value of these practices to provide resources and support [63]. The engagement of multiple school stakeholder groups, including students and parents, is necessary for communicating the nutritional benefits of school meals [64] and improving receptivity to school meals [65]. Similarly to lunch duration, state laws regarding using promotional strategies also predict the use of more of these strategies in schools [26,27].

Consistent with prior literature [66], school personnel in our sample indicated the highest rates of food waste at mealtime came from fruits and vegetables. Over half of schools indicated that entrées, milk, and other beverages were completely or mostly consumed. We found several factors to be associated with less reported food waste, such as using more promotional strategies, which is a unique finding, although it is consistent with the literature regarding the efficacy of multicomponent nutrition interventions in schools for increasing fruit and vegetable consumption [21]. Currently, rigorous research examining the use of promotional strategies specifically in relation to food waste at elementary school lunch (measured independently from selection and consumption) is limited. One study examining the effects of a professional chef’s training of school lunch staff to cook and promote healthy meal choices showed no difference in waste, while improving the health of school meals [25]. Another multiyear intervention showed decreased fruit and vegetable waste as a result of teacher and parent education, as well as hands-on food preparation classes for elementary students [37]. A recent systematic review found that nudge interventions that take place in the cafeteria environment (e.g., point of sale marketing, salad bar placement) showed inconsistent relationships with food consumption and food waste [67]. The limited literature regarding school meal consumption and waste in relation to promotion and education strategies points to the need for more research in this area. 

Having mid-day recess neither directly before nor after lunch was also associated with less food waste in our data. However, it should be noted that few schools reported this scheduling, and this group of schools on average reported a longer lunch duration (28 min versus 24.5 min for both the ‘lunch before recess’ and ‘recess before lunch’ groups), which may have yielded a biased estimate for this group. Although we did not find that lunch duration was significantly associated with overall estimated food waste, contrasting with previous literature [19,30,31], these findings could indicate that schools have more success providing longer lunch times when recess is not directly adjacent to lunch time, which warrants further consideration for scheduling.

In 2018, the USDA rolled back several nutritional guidelines, citing the need for flexibilities for schools to comply with Healthy, Hunger-Free Kids Act recommendations [68] and to encourage student participation. These changes in rules were not supported by evidence based on USDA data [69] and other research [70] that found a vast majority of schools across all states in the US were in compliance with the new guidelines. Further, participation in school meals did not drop after the implementation of the Healthy, Hunger-Free Kids Act [46]. Our findings also show that respondents at very few schools (about one in five) reported using relaxed nutritional guidelines for school meals, and even fewer (< 5%) reported specific changes to offerings that reflected use of the rolled back standards, although many respondents indicated that they did not know about this topic.

### Limitations

Survey responses are subject to social desirability bias, and therefore may not be reflective of actual practices within a school. Although some of the survey items used in this study had previously been used by our team, others were developed specifically for this study and were not pilot-tested; our team includes researchers with extensive experience studying US school nutrition policies and practices, and the items have face validity. However, the lack of data on item validity, reliability, and pretesting for comprehension by target respondents is a limitation. School personnel who completed the survey may have had less complete knowledge of cafeteria-related practices than food service staff, who were not explicitly asked to complete the section of the survey related to nutrition practices. Additionally, our food waste measure warrants further consideration. Although analyses showed appropriate inter-item reliability across the various items pertaining to food waste, the interpretation of food waste using this measure is novel, and other strategies such as visual inspection, digital photography, and plate weighing, conducted over multiple time periods, provide more valid and reliable estimates of food waste for lunch service than a single self-report based on estimation [66]. It is likely challenging for respondents to assess a construct such as ‘waste’ over multiple days for multiple students, accounting for differences in school menus across days, as well as differences in individual consumption habits. In addition, many respondents (49.5%) did not know whether USDA flexibilities had been used, so it is likely that this is a topic that instead should be investigated with district-level administrators/supervisors of food service departments, who are more familiar with regulatory issues than building-level administrators such as principals, or other school staff. This study examined cross-sectional associations between school meal practices and food waste outcomes; therefore, no causal inferences can be made. To examine the impact of practices such as midday recess timing and meal promotion strategies on changes in food waste, future studies should consider randomized controlled trials. While our study was not an intervention trial, the use of a fairly large, nationally-representative sample of elementary schools allows for inference regarding the prevalence of practices pertaining to SBP and NSLP, as well as factors related to food waste, at elementary schools across the US.

## 5. Conclusions

Results from this nationally representative sample show that public elementary schools in the US have increased the adoption of some evidence-based practices for meal service in the last several years. Over 90% of schools that participate in the NSLP also offer breakfast through the SBP, and more are using flexible breakfast service strategies, such as grab-and-go meals, than has been previously reported. However, providing students with at least 30 min for eating lunch, and providing recess before lunch, are both recommended strategies that are used by less than half of schools, and the adoption of these strategies appears to have remained relatively stable over the last few years. Additionally, school meals appear likely to have remained stable in nutrition quality in school year 2019–20, despite regulatory rollbacks in 2018 that have since been rescinded.

Strategies to promote school meals are underutilized, with schools favoring lower-cost strategies that target parents (e.g., newsletters) more often than resource-intensive strategies such as cooking classes and student advisory groups. The use of more promotional strategies was found to be associated with less reported lunch waste, which is a unique finding warranting further investigation. Overall, these findings are consistent with prior literature demonstrating that barriers such as scheduling logistics and resources likely interfere with schools’ implementation of some evidence-based practices (e.g., longer lunch periods and promotion of meals). However, schools have been successful in increasing their use of alternative breakfast service strategies, which represents an important advance in improving access to nutrition assistance programs for socioeconomically disadvantaged students.

## Figures and Tables

**Table 1:** Demographic characteristics of 542 US public elementary schools, 2019–20 school year.

Variable Name	Number (Unweighted)	Percentage (Weighted)
School size		
Small (≤450)	270	49.9
Medium (>450 to 621 students)	165	28.8
Large (>621 students)	107	21.3
Socioeconomic status (% of students eligible for FRPL)		
Higher (≤33%)	144	22.3
Middle (>33% to ≤66%)	209	36.6
Lower (>66%)	188	41.1
Locale		
Urban	110	31.5
Suburban	195	36.8
Township	79	10.2
Rural	158	21.6
US Census Region		
Northeast	104	15.7
Midwest	164	24.4
South	193	35.3
West	81	24.6
Racial/ethnic composition of students		
Predominantly (≥66%) White non-Latino	244	34.3
Majority (≥50%) Black non-Latino	43	8.9
Majority (≥50%) Latino	83	21.6
Other majority or diverse	172	35.3

Note: FRPL = free/reduced-price lunch.

**Table 2:** Descriptive statistics for breakfast and lunch practices at 542 US public elementary schools, 2019–20 school year.

	%	95% CI
Participates in School Breakfast Program		
Yes	91.9	88.9, 94.1
No	8.1	5.9, 11.1
Breakfast service strategy (check all that apply) ^ǂ^		
Traditional/in cafeteria	74.4	69.8, 78.6
Breakfast in the classroom	30.4	25.8, 35.5
Second-chance breakfast	12.0	8.7, 16.4
Grab-and-go breakfast	23.1	19.3, 27.4
Breakfast service strategy combinations ^ǂ^		
In cafeteria only	44.3	39.1, 49.5
Cafeteria + at least 1 other strategy	26.0	22.1, 30.4
Only alternative strategies	24.1	20.2, 28.5
School serves breakfast but answer missing	5.6	3.4, 9.1
Lunch duration for third grade classes		
20 or fewer minutes	37.6	32.0, 43.4
21–29 min	23.2	19.2, 27.6
30+ min	39.3	33.9, 44.9
Timing of lunch to mid-day recess for third grade classes		
Lunch then recess	52.8	47.6, 57.9
Recess then lunch	25.4	20.9, 31.5
Recess is not directly before/after lunch	11.3	8.6, 14.6
Varies by class	10.5	7.7, 14.2

Note: Weighted prevalence estimates may not sum to 100% due to rounding. ^ǂ^ Analyses limited to subsample of 476 schools that used the School Breakfast Program in 2019–20.

**Table 3:** Survey responses regarding meal flexibilities/modifications and strategies used to promote school meals at 542 US public elementary schools, 2019–20 school year.

	Yes		No		Don’t Know	
Survey Item	N	%	N	%	N	%
Used relaxed nutrition standards	112	21.0	156	29.4	249	49.5
	Less/Fewer		Same		More	
Meal modifications since last year	N	%	N	%	N	%
Fruit/vegetables: amount	8	1.5	404	75.8	104	22.7
Fruit/vegetables: variety	12	2.0	369	69.1	135	28.9
Wholegrain options	23	3.3	417	80.2	76	16.4
Low-fat dairy options	6	0.8	455	87.6	55	11.6
Variety of entrée options	22	4.0	379	72.3	115	23.7
	Never		1–2 Times		3+ Times	
Promotional strategies used for meals	N	%	N	%	N	%
Student taste tests	326	66.3	133	25.3	49	8.4
Student advisory groups	406	81.9	81	16.1	11	2.0
Cooking club demonstrations/classes	424	84.7	59	11.4	17	3.9
Social media (Facebook, Twitter, etc.)	353	72.6	103	19.1	45	8.3
Engagement with PTA or parent groups	335	66.0	135	27.7	33	6.3
Newsletters	254	50.1	171	32.3	86	17.6

Note. Estimates may not sum to 100% due to rounding. N = number (unweighted), % = percentage (weighted).

**Table 4:** Perception of amount of school lunch components that students eat (i.e., not wasted), at 542 US public elementary schools, 2019–20 school year.

Amount Eaten	All		Most		Some		A Little		None	
	N	%	N	%	N	%	N	%	N	%
School lunch										
Entrée	39	7.7	322	60.1	118	24.5	32	7.1	3	0.6
Fruit	24	4.4	244	48.4	193	37.7	50	8.9	3	0.6
Vegetable	10	2.2	117	22.8	274	54.4	109	19.9	5	0.8
Milk	69	12.7	278	54.7	113	22.8	47	9.0	5	0.8

Note. Estimates may not sum to 100% due to rounding. N = number (unweighted), % = percentage (weighted).

**Table 5:** Regression models predicting perceived extent of food waste in school lunches.

	Waste—Overall ^⸙^			Waste—Composite (Entrée, Milk, Fruit) ^⸙⸙^			Waste—Vegetables ^⸙⸙^		
Predictor Variables	Coeff.	SE	*p*	Coeff.	SE	*p*	Coeff.	SE	*p*
Number of promotional strategies (0 to 6)	**−0.063**	**0.025**	**0.013**	**−0.075**	**0.019**	**<0.001**	−0.011	0.022	0.625
≥30 Min lunch duration (vs <30 min)	−0.119	0.078	0.131	−0.008	0.062	0.897	−0.083	0.078	0.283
Mid-day recess timing									
After lunch (referent)	-			-			-		
Before lunch	0.047	0.083	0.575	−0.139	0.077	0.072	−0.006	0.093	0.946
Neither directly before or after lunch	0.057	0.105	0.585	**−0.221**	**0.082**	**0.007**	−0.172	0.123	0.162
Timing varies by class	−0.328	0.206	0.111	−0.099	0.135	0.465	−0.020	0.126	0.875
School size									
Small (≤450; referent)	-			-			-		
Medium (>450 to 621 students)	−0.104	0.092	0.258	−0.076	0.072	0.293	0.064	0.075	0.396
Large (>621 students)	0.112	0.109	0.303	−0.113	0.099	0.253	−0.146	0.116	0.208
Socioeconomic status (% eligible for FRPL)									
Higher (≤33%; referent)	-			-			-		
Mid (>33% to ≤66%)	0.053	0.089	0.549	−0.017	0.078	0.831	−0.021	0.090	0.815
Lower (>66%)	−0.216	0.120	0.072	0.070	0.104	0.502	−0.203	0.118	0.086
Locale									
Urban (referent)	-			-			-		
Suburban	0.049	0.101	0.628	−0.023	0.079	0.771	**0.204**	**0.087**	**0.020**
Township	−0.025	0.132	0.852	**−0.267**	**0.097**	**0.006**	0.139	0.138	0.315
Rural	0.047	0.112	0.677	−0.041	0.101	0.681	**0.298**	**0.114**	**0.009**
Region									
Northeast (referent)	-			-			-		
Midwest	−0.052	0.105	0.621	−0.068	0.087	0.437	−0.057	0.100	0.567
South	−0.048	0.115	0.678	−0.035	0.103	0.731	0.099	0.097	0.308
West	0.026	0.126	0.839	0.051	0.122	0.673	−0.071	0.118	0.547
Student racial/ethnic composition									
Predominantly (≥66%) White non-Latino (referent)	-			-			-		
Majority (≥50%) Black non-Latino	0.083	0.205	0.686	−0.149	0.154	0.333	0.180	0.183	0.326
Majority (≥50%) Latino	0.118	0.142	0.406	0.034	0.128	0.792	0.330	0.135	0.015
Other majority or diverse	−0.105	0.100	0.296	0.021	0.081	0.793	0.186	0.097	0.057
Number of schools	484			479			481		

Note. FRPL = Free/reduced price lunch. ^⸙^ Outcome reverse-coded to reflect extent to which food waste is a problem, 0 = none to 3 = very much. ^⸙⸙^ Outcomes reflect perceptions of amounts eaten; higher values reflect more waste (1 = all eaten to 5 = none eaten). Estimates highlighted in bold are significant at *p* < 0.05 or better.

## Data Availability

To request access to de-identified data, please contact the corresponding author.
